# Multidrug-Resistant Bacteria in Children and Adolescents with Cystic Fibrosis

**DOI:** 10.3390/children9091330

**Published:** 2022-09-01

**Authors:** Valentina Fainardi, Cosimo Neglia, Maria Muscarà, Cinzia Spaggiari, Marco Tornesello, Roberto Grandinetti, Alberto Argentiero, Adriana Calderaro, Susanna Esposito, Giovanna Pisi

**Affiliations:** 1 Pediatric Clinic, University Hospital, Department of Medicine and Surgery, University of Parma, 43126 Parma, Italy; 2 Department of Medicine and Surgery, University of Parma, 43126 Parma, Italy

**Keywords:** antimicrobial resistance, cystic fibrosis, multidrug-resistant bacteria, pulmonary exacerbation, *Stenotrophomonas maltophilia*

## Abstract

In patients with cystic fibrosis (CF), multidrug-resistant (MDR) bacteria can predispose to exacerbations, limit the effectiveness of antibiotic treatments and promote the progression of lung disease. The aim of this retrospective study was to compare pulmonary exacerbations (Pex), hospitalizations, lung function and nutritional status in a group of children and adolescents with CF colonized by MDR bacteria and in a noncolonized control group. Overall, 7/54 pediatric patients (12.9%) were colonized by MDR bacteria and enrolled (3 with *Achromobacter xyloxidans*, 3 with *Stenotrophomonas maltophilia* and 1 with *Burkholderia cepacia*). The control group included 14 sex- and age-matched CF patients (8/14 colonized by *Staphylococcus aureus,* 2/14 by *Pseudomonas aeruginosa*, 2/14 by both microorganisms and 2/14 germ free). At the time of enrollment and 12 months before the first detection of the MDR microorganism, children colonized by MDR bacteria showed lower body mass index (BMI) and lower FEV_1_/FVC compared to the control group. Over the previous year before the first detection, children colonized with MDR had more Pex compared to control group; those colonized by *S. maltophilia* experienced the highest number of Pex. In the 12 months following the first detection of MDR bacteria, all seven patients colonized by MDR had at least one Pex and patients colonized by *S. maltophilia* had the highest number (mean ± SD: 6 ± 2.6 vs. 1.7 ± 2.3). Our study suggests that CF pediatric patients infected by MDR bacteria have lower BMI, more obstructive disease and experience more exacerbations than patients without MDR bacteria. These differences are present even before being infected, suggesting that children and adolescents with more severe disease are predisposed to be colonized by MDR bacteria. *S. maltophilia* appeared to be the most aggressive pathogen. Further studies and the implementation of antimicrobial stewardship programs are necessary to clarify when and how to treat patients with CF and MDR bacteria in order to avoid the improper use of antibiotics and the development of antibiotic resistance.

## 1. Introduction

Cystic fibrosis (CF) is the most common autosomal-recessive life-limiting disease in European countries. In airway epithelial cells, the defective activity of cystic fibrosis transmembrane conductance regulator (CFTR) is associated with abnormal sodium chloride and water transport that leads to the accumulation of viscous secretions and impaired ciliary clearance [1]. This condition favors colonization and chronic infection by opportunistic pathogens with persistent respiratory infections and progressive worsening of lung function. The survival of individuals with CF has dramatically increased over time thanks to improvements in their general care, including airway clearance techniques, better nutrition, antibiotic treatments [2] and, more recently, modulator compounds able to enhance and regulate the activity of CFTR [3]. However, the emergence of multidrug-resistant (MDR) microorganisms predispose to exacerbations, limit the effectiveness of antibiotic treatments and promote progression of lung disease [4].

In CF, the widespread use of systemic antibiotics to treat exacerbations or eradicate *Pseudomonas aeruginosa* and the chronic administration of inhaled antibiotics to suppress or control rather than kill bacteria are partly the cause of this antimicrobial resistance (AMR). AMR is a significant challenge in the treatment of lung infections [5] and the recent constitution of the International Working Group on AMR in CF [6] aims to address this issue. *Stenotrophomonas maltophilia*, *Achromobacter xyloxidans* and *Burkholderia cepacia*, all ubiquitous environmental bacteria, show a broad spectrum of intrinsic and acquired mechanisms of AMR, which makes eradication difficult [7]. At present, there are no clear indications on eradication timing or on the preferred antibiotic therapy in acute and chronic conditions. Furthermore, there is a well-described discordance between clinical outcomes and antimicrobial susceptibility test (AST) results in individuals with CF [8]. The aim of the present study was to compare pulmonary exacerbations (Pex), hospitalizations, nutritional status and pulmonary function testing (PFT) in pediatric patients with CF colonized by MDR bacteria and in a noncolonized control group.

## 2. Methods

### 2.1. Study Design and Population

This is an observational, retrospective, nonprofit study conducted at the CF Center of Parma University Hospital (Italy). CF patients aged ≤ 18 years and colonized by MDR pathogens (*S. maltophilia*, *A. xyloxidans, B. cepacia*, atypical *Mycobacteria,* *Prevotella intermedia*) were included in the study (MDR group); data collection was performed in January 2021. Data included age, gender, genotype and presence of pancreatic insufficiency; microbiological results of sputum culture (including antibiograms) and the mean values of body mass index (BMI), episodes of Pex, number of hospitalizations, antibiotic courses and PFTs (a) over the year before data collection (i.e., year 2021); (b) over the 12 months before the first detection of the MDR bacteria; (c) over the 12 months after the first detection of the MDR bacteria were also collected. Colonization was defined when sputum culture was positive for MDR bacteria in more than 50% of samples in 12 months. Patients not colonized by MDR bacteria matched for age and sex were enrolled as controls; each patient with MDR bacteria had two matched children as controls (control group). Subjects with lung transplantation, severely immunocompromised patients, tumor of solid organ or haematological malignancy were excluded from the study. The study was approved by Ethical Committee of Emilia-Romagna Area Vasta Nord. Before inclusion, parents signed their written informed consent and patients signed their written assent.

### 2.2. Statistical Analysis

Data analysis was performed using STATA Statistical Software (Release 11 College Station, TX, USA). For the descriptive analysis of the continuous variables (age, BMI, Pex, number of hospitalizations, PFT) the mean and standard deviation (SD) were calculated. The dichotomous and qualitative variables are reported as absolute frequency and percentage frequency. For the comparison of mean values of the continuous variables in MDR and control groups, Student’s t-test was used. A *p* < 0.05 value was considered as statistically significant.

## 3. Results

Of the 54 pediatric patients with CF followed in our Center, the study included 7 patients (12.9%, 7 M) colonized by MDR bacteria. The first finding of the MDR bacteria in the sputum occurred at an average age of 10.7 ± 2.2 years. Three patients (5.5%) showed *A. xyloxidans*, three showed (5.5%) *S. maltophilia* and one showed *B. cepacia* (1.8%). None were colonized by atypical *Mycobacteria* and *Prevotella intermedia*. The control group included 14 matched CF patients: 8/14 (57.1%) colonized by *S. aureus*, 2/14 (14.3%) by *P. aeruginosa*, 2/14 (14.3%) by both microorganisms and 2/14 (14.3%) were germ-free. At the time of data collection, patients colonized by MDR bacteria showed mean values of BMI and forced expiratory flow in 1 s (FEV_1_)/forced vital capacity (FVC) ratio significantly lower than control group. The comparison of characteristics and PFT between MDR group and control group at the time of data collection is shown in Table 1.

In the year before the first detection of MDR bacteria, patients later colonized by MDR bacteria had significantly more Pex (mean ± SD: 5 ± 2.5 vs. 1.7 ± 2.3, t = 3.28, *p* < 0.003) than the control group, with 4 out of 7 (57.1%) requiring hospitalization. Patients with *S. maltophilia* showed the highest number of Pex (mean ± SD: 3.3 ± 1.5 vs. 1.7 ± 0.9, *p* < 0.02). In the control group, 2/14 (14.3%) patients were hospitalized. Moreover, the MDR group had mean values of BMI and FEV_1_/FVC ratio significantly lower than the control group. Characteristics and PFT of the MDR group over the year before the first detection of the MDR bacteria are reported in Table 2.

In the 12 months following the first detection of MDR bacteria, the difference in FEV_1_/FVC ratio, BMI and Pex between patients colonized by MDR and controls persisted (Table 3). In addition, all seven patients colonized by MDR had at least one Pex (mean ± SD: 3.5 ± 3.2), with two patients (28.5%) requiring hospitalization. Patients colonized by *S. maltophilia* had the highest number of Pex (mean ± SD: 6 ± 2.6 vs. 1.7 ± 2.3). At the time of the first finding of the MDR bacteria (mean age ± SD: 10.7 ± 2.2 years), four patients were immediately treated with a course of 14 days of oral or systemic antibiotics.

## 4. Discussion

This study showed that children and adolescents with CF colonized by MDR bacteria have a significantly greater number of pulmonary exacerbations, worse nutritional status and more obstructive lung condition before colonization compared to those not colonized by MDR bacteria. As expected, these differences between the two groups significantly persisted after the colonization by MDR bacteria.

In the present study, we found that of the 54 children with CF followed in our Center, 12.9% were colonized by MDR bacteria and the first finding occurred at an average age of 10.7 years. The average age of the first finding found in our population was very similar to the average age reported by an Italian study of 300 CF young patients (10.7 years vs. 11.5 years) [9]. Three patients (5.5%) showed in *A. xyloxidans* in the sputum, three (5.5%) showed *S. maltophilia* and one showed *Burkholderia cepacia* (1.8%). Prevalent studies of *A. xyloxidans* colonization and chronic infection in CF patients varied greatly with rates ranging from 3 to 30% [10,11,12]. Similarly, rates of *S. maltophilia* colonization in CF patients varied considerably from Center to Center (from 0 to 30%) [13] and most of the isolates appeared to be transient, resulting in persistent infection in 13–23% of cases [14,15].

At the time of the first finding of the MDR bacteria, four patients in our study population were immediately treated with a course of 14 days of oral or systemic antibiotics with disappointing results. However, whether chronic infection by MDR can be prevented with eradication is still unknown [16]. Concerning *A. xyloxidans*, the occurrence of MDR is common and there are no data on optimal treatment strategies in CF. Guidelines based on expert opinions suggest that antibiogram-directed therapy should be offered [17]. Antimicrobial resistance is a characteristic of *S. maltophilia* as well. Treatment data for *S. maltophilia* are also lacking in CF, and eradication strategies have not been pursued [18]. Trimethoprim/sulfamethoxazole plus an additional antimicrobial agent are recommended by expert panels to treat Pex in *S. maltophilia*-colonized patients [18].

The prevalence of *Burkholderia cepacia* in our population was similar to that reported in the study by Lambiase et al. (1.8% vs. 0.07%), while our prevalence of *S. maltophilia* and *A. xyloxidans* infection was lower (5.5% vs. 18.9% and 5.5% vs. 17.6%, respectively) [9]. Differently from other authors [19], we did not notice any significant seasonality in the colonization by MDR bacteria.

In our study, all CF patients colonized by MDR bacteria were males. Nevertheless, female sex in CF was shown to be a significant risk factor for death; women also acquire *P. aeruginosa* at an earlier age, as well as several other pathogens such as *S. aureus*, *Haemophilus influenzae*, *A. xyloxidans*, *B. cepacia*, *Aspergillus* and nontuberculous mycobacteria [20]. A wide range of factors have been examined to explain the sex gap in airway diseases [21]. In a study investigating the impact of inhaled aztreonam on the lung microbiome in CF, males showed a significantly higher Shannon diversity index with lower abundance of *P. aeruginosa* and increased abundance in other genera, including *Streptococcus* spp. and *Stenotrophomonas* spp. In contrast, females showed a higher abundance of *Pseudomonas* spp. [22]. In a retrospective cohort, Chotirmall et al. found that nonmucoid *P. aeruginosa* predominated in sputum during luteal phase exacerbations (characterized by low estradiol rates) and that more mucoid *P. aeruginosa* strains were isolated during Pex in the follicular phase (with high estradiol rates) [23]. These results are consistent with other in vitro studies showing that estrogen increased secretion of *P. aeruginosa* virulence factors, biofilm formation and invasion of bronchial epithelial cells [24].

Both in the twelve months before the isolation of the MDR bacteria and in the following year, patients with MDR bacteria had significantly more Pex than controls. Notably, in both cases in the MDR colonized group of patients, those with *S. maltophilia* chronic infection had the highest number of Pex. The most severe clinical picture of patients colonized by MDR bacteria both at the time of data collection and one year before the first isolation of MDR bacteria suggests that these subjects might be predisposed to be colonized by MDR bacteria because of a most severe form of CF disease. MDR bacteria colonization might further contribute to the clinical deterioration, but the exact contribution cannot be extrapolated from these data.

Compared to controls, children colonized by MDR bacteria showed a lower FEV_1_/FVC ratio in all time points, suggesting a condition of obstructive disease. Between the first detection of the bacteria and data collection (over a period of 3 ± 1.5 years), subjects colonized by MDR bacteria experienced a greater decline in lung function compared to controls (11.1% vs. 7.5%). The finding of lower lung function in patients with MDR bacteria was reported by several studies. Dalbøge et al. found that patients with chronic *S. maltophilia* infection had an increased rate of decline in FEV_1_ in comparison with controls, and this rate of decline was higher than that of controls prior to the development of *S. maltophilia* [25]. Recently, retrospective data suggested that children with more severe Pex are at higher risk of *S. maltophilia* infection in the following year and showed a worse clinical outcome compared to controls [26]. However, in a cohort study conducted by the CF Foundation National Patient Registry in more than 20,000 patients, no increased decline in lung function nor any survival impact was observed with *S. maltophilia* infection [27,28]. More recent data showed that chronic infection by *S. maltophilia* was associated with increased risk of Pex and death/transplantation [29,30].

The impact of *A. xyloxidans* on outcomes in CF is yet unclear. Patients chronically colonized by *A. xyloxidans* showed lower FEV_1_, more rapid decline of pulmonary function and higher rate of Pex [31,32,33]. Similarly, De Baets et al. demonstrated that patients chronically infected with *A. xyloxidans* were more likely to have lower lung function and worse radiographs but did not experience an increased burden of Pex [12]. This finding suggests that patients with increased burden of structural lung disease are predisposed to infection.

The main limitations of our study are its retrospective design and the small number of the enrolled patients, although they are partially balanced by suitable controls. However, it showed the significant clinical impact of MDR bacteria in pediatric patients with CF and highlighted the importance of developing a standardized protocol for their management.

## 5. Conclusions

Our study suggests that children and adolescents with CF colonized by MDR bacteria experience more exacerbations and have more obstructive lung disease and lower BMI than controls even before being colonized. We can speculate that children and adolescents with advanced disease might be predisposed to be colonized by MDR bacteria because of a more severe form of CF disease. Among the MDR bacteria, *S. maltophilia* appears to be the most aggressive in terms of exacerbations. Both a severe form of CF and MDR bacteria colonization may contribute to deterioration and lung function decline. With the introduction of new antibiotics and novel diagnostic technologies, a new generation of emerging bacteria can be expected. Whether these pathogens will be colonizers or associated with clinical deterioration should be understood. Further studies and the implementation of antimicrobial stewardship programs are necessary to clarify when and how to treat them in order to avoid the improper use of antibiotics and further negative effects on the development of antibiotic resistance.

## Figures and Tables

**Table 1:** Characteristics and pulmonary function tests at time of data collection in patients with CF colonized by MDR bacteria and in control group.

	Patients Colonized with MDR Bacteria (*n* = 7)	Matched Controls (*n* = 14)
Age (years)	14.2 ± 1.8	14.3 ± 3.9
Genotype (F/F, *n*)	2/7	2/14
BMI (kg/m^2^)	16.9 ± 1.6	19.9 ± 3.4 *
FEV_1_ pp %	76.5 ± 27.0	88.7± 21.3
FVC pp	88.1 ± 26.2	92.8 ± 14.7
FEV_1_/FVC	75.7 ± 9.6	82.6 ± 10.1 **
Pex	2 ± 1.6	0.3 ± 0.7 **

Data are expressed as mean ± SD. BMI, body mass index; FEV_1_, forced expiratory flow in 1 s; FVC, forced vital capacity; pp, percent of predicted; F/F, homozygous F508 del; Pex, pulmonary exacerbations. * t = 4.29, *p* < 0.001; ** t = 2.19, *p* < 0.03.

**Table 2:** Characteristics and pulmonary function tests in the year before the first detection of MDR bacteria in MDR group and in control group.

	Patients Colonized with MDR Bacteria (*n* = 7)	Matched Controls (*n* = 14)
Age (years)	10.3 ± 2.2	10.6 ± 3.9
BMI (kg/m^2^)	15.6 ± 1.9	19.2 ± 4.5 *
FEV_1_ pp %	87.6 ± 15.5	93.2 ± 12.8
FVC pp %	101.0 ± 11.4	96.2 ± 13.3
FEV_1_/FVC %	75.8 ± 9.4	87.0 ± 5.2 **
Pex	5 ± 2.5	1.7 ± 2.3 *

Data are expressed as mean ± SD. BMI, body mass index. FEV_1_, forced expiratory flow in 1 s; FVC, forced vital capacity; pp, percent of predicted; Pex, pulmonary exacerbations. * t = 2.06, *p* < 0.05; ** t = 3.58, *p* < 0.002.

**Table 3:** Characteristics and pulmonary function tests in the year following the first detection of MDR bacteria in MDR group and in control group.

	Patients Colonized with MDR Bacteria (*n* = 7)	Matched Controls (*n* = 14)
Age (years)	12.1 ± 1.4	11.9 ± 4.0
BMI (kg/m^2^)	16.3 ± 2.5	18.6 ± 2.3 **
FEV_1_ pp %	85.4 ± 14.6	88 ± 13.2
FVC pp %	95.0 ± 13.4	90.3 ± 11.3
FEV_1_/FVC %	77.7 ± 10.0	95.8 ± 7.8 **
Pex	3.5 ± 3.2	1 ± 1.5 *

Data are expressed as mean ± SD. BMI, body mass index; FEV_1_, forced expiratory flow in 1 s; FVC, forced vital capacity; pp, percent of predicted; Pex, pulmonary exacerbations. * t = 2.06, *p* < 0.05; ** t = 3.58, *p* < 0.002.

## Data Availability

The data presented in this study are available on request from the corresponding author.
